# Metformin Causes G1-Phase Arrest via Down-Regulation of MiR-221 and Enhances TRAIL Sensitivity through DR5 Up-Regulation in Pancreatic Cancer Cells

**DOI:** 10.1371/journal.pone.0125779

**Published:** 2015-05-08

**Authors:** Ryoichi Tanaka, Mitsuhiro Tomosugi, Mano Horinaka, Yoshihiro Sowa, Toshiyuki Sakai

**Affiliations:** Department of Molecular-Targeting Cancer Prevention, Graduate School of Medical Science, Kyoto Prefectural University of Medicine, Kyoto, Japan; Winship Cancer Institute of Emory University, UNITED STATES

## Abstract

Although many chemotherapeutic strategies against cancer have been developed, pancreatic cancer is one of the most aggressive and intractable types of malignancies. Therefore, new strategies and anti-cancer agents are necessary to treat this disease. Metformin is a widely used drug for type-2 diabetes, and is also known as a promising candidate anti-cancer agent from recent studies *in vitro* and *in vivo*. However, the mechanisms of metformin’s anti-cancer effects have not been elucidated. We demonstrated that metformin suppressed the expression of miR-221, one of the most well-known oncogenic microRNAs, in human pancreatic cancer PANC-1 cells. Moreover, we showed that the down-regulation of miR-221 by metformin caused G1-phase arrest via the up-regulation of p27, one of the direct targets of miR-221. Tumor necrosis factor-related apoptosis-inducing ligand (TRAIL) is also a promising agent for cancer treatment. While recent studies showed that treatment with only TRAIL was not effective against pancreatic cancer cells, the present data showed that metformin sensitized p53-mutated pancreatic cancer cells to TRAIL. Metformin induced the expressions of death receptor 5 (DR5), a receptor for TRAIL, and Bim with a pro-apoptotic function in the downstream of TRAIL-DR5 pathway. We suggest that the up-regulation of these proteins may contribute to sensitization of TRAIL-induced apoptosis. The combination therapy of metformin and TRAIL could therefore be effective in the treatment of pancreatic cancer.

## Introduction

Pancreatic cancer is a refractory cancer and the fourth-leading cause of cancer death in the United States [1]. The only curative treatment for this malignant tumor is surgery and the five-year relative survival of patients with pancreatic cancers was 2–6% in the United States from 1975 to 2009. Gemcitabine was established as first-line chemotherapy in the 1990s [2]. FOLFIRINOX (oxaliplatin, irinotecan, leucovorin, and fluorouracil) or combination therapy of gemcitabine and erlotinib, a selective inhibitor of EGFR tyrosine kinase, partially improved overall survival, but not enough [2–4]. Therefore, more effective drugs or combination therapies for pancreatic cancers are needed.

Metformin has been widely used as a drug for type 2 diabetes for a long time [5]. Today, metformin is considered the first choice for oral treatment for type 2 diabetes because there are no major contraindications and the cost of the drug is low [6]. Meanwhile, recent reports have shown that metformin is useful in cancer prevention and treatment [7]. Several clinical studies of metformin in patients with cancers are ongoing [8, 9]. Metformin decreases glucose production in the liver, activates the liver kinase B1 (LKB1)/AMP kinase (AMPK) axis and inhibits the mammalian target of rapamycin complex 1 (mTORC1). It also inhibits insulin growth factor-1 (IGF-1) [10–13]. Moreover, metformin regulates several microRNA expressions [14] and targets cancer stem cells [12, 15]. A treatment with metformin inhibited the growth of cancer cells by inducing G1-phase arrest via up-regulation of p27 [16]. However, the precise mechanisms by which metformin up-regulates p27 remain unclear.

Tumor necrosis factor-related apoptosis-inducing ligand (TRAIL/Apo2L) induces apoptosis not in normal cells, but selectively in malignant tumor cells [17–19]. Recombinant human TRAIL and agonistic antibodies for TRAIL receptors are attractive anti-cancer agents and several TRAIL-based clinical trials are underway [20]. TRAIL induces apoptosis in various cancer cells via death receptor 5 (DR5; also called TRAIL-R2), one of the five TRAIL receptors [21–23]. However, there is an important problem that some pancreatic cancer cells are insensitive to TRAIL-mediated apoptosis [24, 25]. Recently, specific microRNAs have been reported to be related to the resistance of TRAIL in cancer cells [26]. MicroRNAs are a class of small noncoding RNAs that regulate target gene expressions by translational repression and mRNA cleavage. MicroRNAs have been demonstrated to play an important role in the process of carcinogenesis [27]. The role of microRNAs has been studied in many types of tumors, including pancreatic cancers. Among them, miR-221 is involved in tumor development by regulating cell proliferation and it contributes to TRAIL resistance [28–31]. The expression of miR-221 is increased in human pancreatic cancer cells [32]. Interestingly, a recent study showed that miR-221 was elevated in the internal mammary arteries of subjects with type 2 diabetes and there was a significant inverse correlation between the oral dose of metformin and the level of miR-221 [33].

A recent study showed that metformin up-regulated DR5 and enhanced the TRAIL sensitivity in p53 wild-type cancer cells [34], which indicates that it is a promising candidate for overcoming TRAIL resistance in cancer cells. However, more than a half of malignant tumors possess inactivating mutations in the p53 gene [35, 36], and therefore we need to examine if metformin enhances the sensitivity to TRAIL in p53-mutant cancer cells.

In the present study, we found that metformin reduced miR-221 expression thereby causing G1-phase arrest through up-regulation of p27, a direct target of miR-221. Furthermore, we showed that metformin enhanced TRAIL sensitivity via up-regulation of DR5 in p53-mutant pancreatic cancer cells.

## Materials and Methods

### Reagents

Metformin was purchased from Sigma (St. Louis, MO, USA). Soluble recombinant human TRAIL/Apo2L was purchased from PeproTech (London, UK). The human recombinant DR5 (TRAIL-R2)/Fc chimera and pan-caspase inhibitor, zVAD-fmk, were purchased from R&D Systems (Minneapolis, MN, USA).

### Cell culture

Human pancreatic cancer PANC-1 and AsPC-1 cells were obtained from the American Type Culture Collection (Rockville, MD, USA). Human pancreatic cancer MIAPaCa-2 cells were purchased from Human Science Research Resources Bank (Osaka, Japan). PANC-1 and MIAPaCa-2 cells were cultured in Dulbecco’s Modified Eagle’s Medium (DMEM) supplemented with 10% fetal bovine serum, 4 mM glutamine, 50 U/mL penicillin, and 100 μg/mL streptomycin. AsPC-1 cells were maintained in RPMI1640 medium supplemented with 10% fetal bovine serum, 2 mM glutamine, 50 U/mL penicillin, and 100 μg/mL streptomycin. All cells were incubated at 37 ^o^C in a humidified atmosphere containing 5% CO_2._

### Cell growth assay

The number of viable cells was determined using the Cell Counting Kit-8 assay according to the manufacturer's instructions (Dojindo Molecular Technology, Kumamoto, Japan). After incubation of cells for 72 hours with the indicated concentrations of metformin or TRAIL, kit reagent WST-8 was added to the medium and it was incubated for a further 4 hours. The absorbance of samples (450 nm) was determined using a scanning multiwell spectrophotometer.

### Trypan blue dye exclusion assay

Cells were plated in 12-well plates (1 x 10^4^ /well). On the next day, cells were treated with metformin at indicated concentrations for 72 hours. Cell viability was measured by the trypan blue dye exclusion assay.

### Analysis of cell cycle progression and detection of apoptosis

Cells were incubated with or without metformin or TRAIL at concentrations indicated and harvested. After washing with PBS, the cells were suspended in PBS containing 0.1% Triton X-100, treated with RNase A (Sigma), and the nuclei were stained with propidium iodide (Sigma). The DNA content was measured using FACS Calibur (Becton Dickinson, Franklin Lakes, NJ, USA). For each experiment, 10,000 cells were analyzed. Cell Quest software (Becton Dickinson) and the ModFit LT V2.0 software package (Verity Software House, Topsham, ME, USA) were used to analyze the data.

### Western blot analysis

Cells were lysed in lysis buffer (50 mM Tris-HCl, 1% SDS, 2 μg/mL leupeptin, 2 μg/mL aprotinin, 0.5 mM PMSF, and 1 mM DTT). The lysate was sonicated and centrifuged at 15,000 g for 20 min at 4°C, and the supernatant was collected. The protein extract was loaded onto a 7.5, 10, or 12.5% SDS-polyacrylamide gel for electrophoresis, and blotted onto polyvinylidene difluoride membranes (Millipore, Bedford, MA, USA). Rabbit polyclonal anti-DR4, anti-DR5 (Prosci, Poway, CA, USA), and anti-CDK4 (Santa Cruz Biotechnology, Santa Cruz, CA, USA), and rabbit monoclonal anti-Bim (Epitomics, San Diego, CA, USA), and mouse monoclonal anti- cyclin D1 (MBL, Nagoya, Japan) and anti-β-actin (Sigma) antibodies were used as the primary antibodies. The blots were incubated with the appropriate HRP-conjugated secondary antibody (GE Healthcare, Piscataway, NJ, USA), and signals were detected using a Chemilumi-one chemiluminescent kit (Nacalai Tesque, Kyoto, Japan).

### Determination of TRAIL receptor expression

Cells were harvested by trypsinization, washed once with ice-cold PBS, and resuspended in 100 μL PBS with 1% BSA. Then, PE-labeled mouse anti-human DR4 or DR5 mAb (eBioscience, San Diego, CA, USA) was added. To assess nonspecific staining, PE-labeled control IgG isotypes (eBioscience) were applied. After a 30 min incubation on ice, 20,000 cells were analyzed by FACSCalibur.

### RNA analysis

Total RNA from the cells was extracted using a mirVana miRNA Isolation kit (Applied Biosystems, Foster, CA, USA), according to the manufacturer’s instructions. For quantitative real-time RT-PCR, total RNA (2 μg) was reverse-transcribed to cDNA in a 20 μL reaction volume using a High Capacity cDNA Reverse Transcription kit (Applied Biosystems) according to the manufacturer’s instructions. TaqMan probes for DR5 and β2-microglobulin (β2MG) were purchased from Applied Biosystems. The expression levels of mRNAs were quantified using the Applied Biosystems 7300 Real-Time PCR system according to the manufacturer’s instructions. The expression level of DR5 mRNA was normalized against the level of β2MG mRNA in the same sample.

microRNA expression analysis was performed by using TaqMan miRNA assays (Applied Biosystems) to evaluate miR-221. Total RNA (10 ng) was reverse-transcribed to cDNA in a 15 μL reaction volume using each specific primers and TaqMan MicroRNA Reverse Transcription kit (Applied Biosystems). The expression levels of microRNAs were quantified using the 7300 Real-Time PCR system (Applied Biosystems) according to the manufacturer’s instructions. The results were normalized relative to the RNA gene RNU48.

### microRNA mimics transfection

Cells were transfected with 5 nM miRIDIAN microRNA Mimics (miR-221 or Negative Control #1; Dharmacon, Lafayette, CO, USA) using Lipofectamine RNAiMAX (Invitrogen, Carlsbad, CA, USA) according to the manufacturer’s instructions. After 24 hours of transfection, cells were treated with or without 40 mM metformin. The cells were harvested 24 hours after the treatment for FACS analysis and Western blotting.

### Statistical Analysis

Data are the means ± S.D. of three determinations. Data was analyzed using the Student’s *t-*test and differences were considered significant at P <0.05.

## Results

### Metformin suppresses the cell growth of pancreatic cancer cells

**Fig 1:**
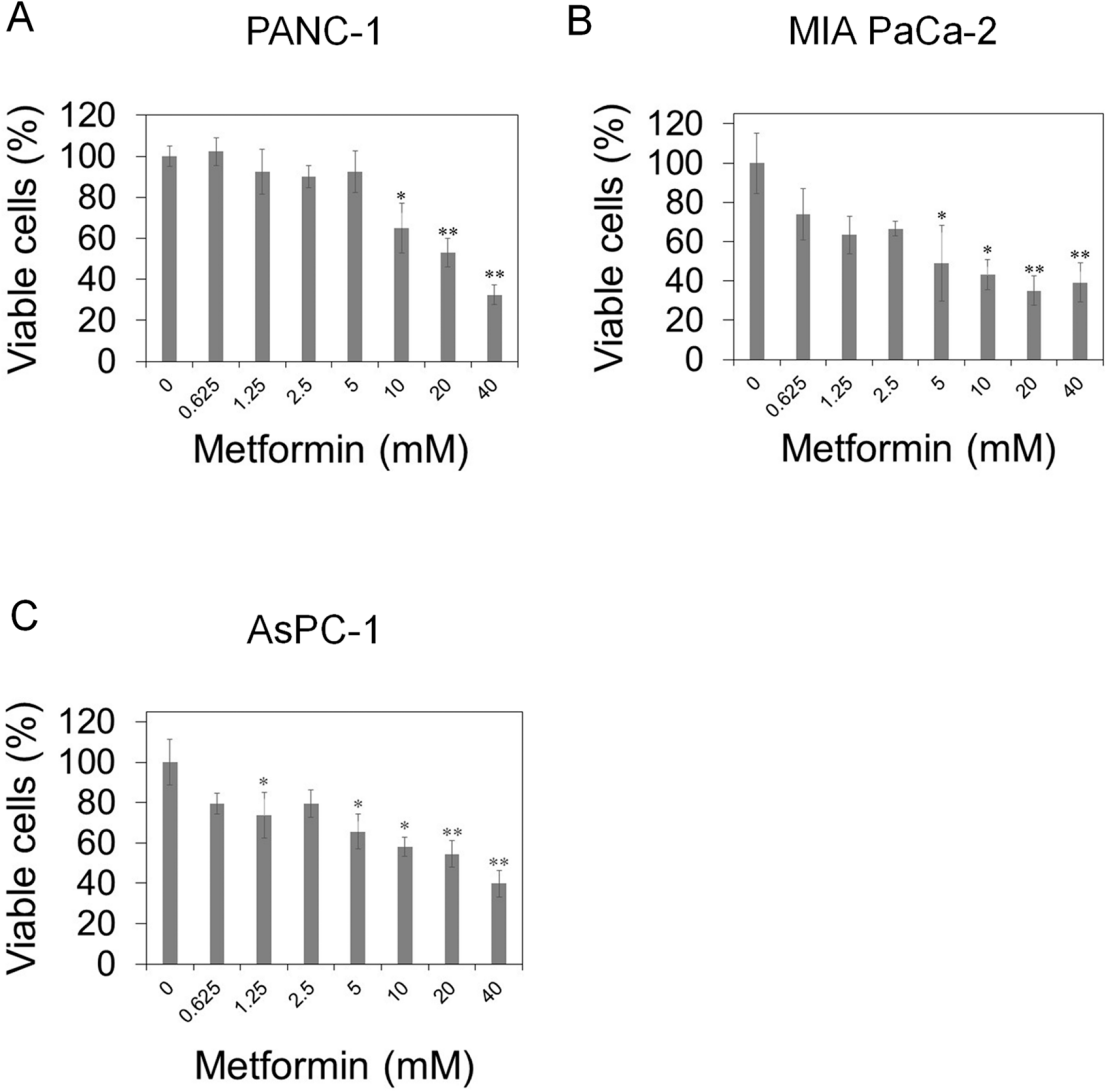
Metformin suppresses the cell growth of pancreatic cancer cells. (A) PANC-1, (B) MIA PaCa-2, and (C) AsPC-1 cells were treated with the indicated concentrations of metformin. After incubation for 72 hours, cells were counted by trypan blue dye exclusion assay. Data are the means ± SD of 3 determinations. *P<0.05, **P<0.01.

To investigate the effect of metformin on human pancreatic cancer cells, we examined whether metformin suppressed the cell growth using trypan blue dye exclusion assay. PANC-1, MIA PaCa-2, and AsPC-1 cells were treated with indicated concentrations of metformin for 72 hours, and viable cells were counted by trypan blue dye exclusion assay. As shown in Fig 1, metformin decreased the growth of these cancer cells in a dose-dependent manner.

### Metformin induces G1-phase arrest in pancreatic cancer cells through down-regulation of miR-221

**Fig 2:**
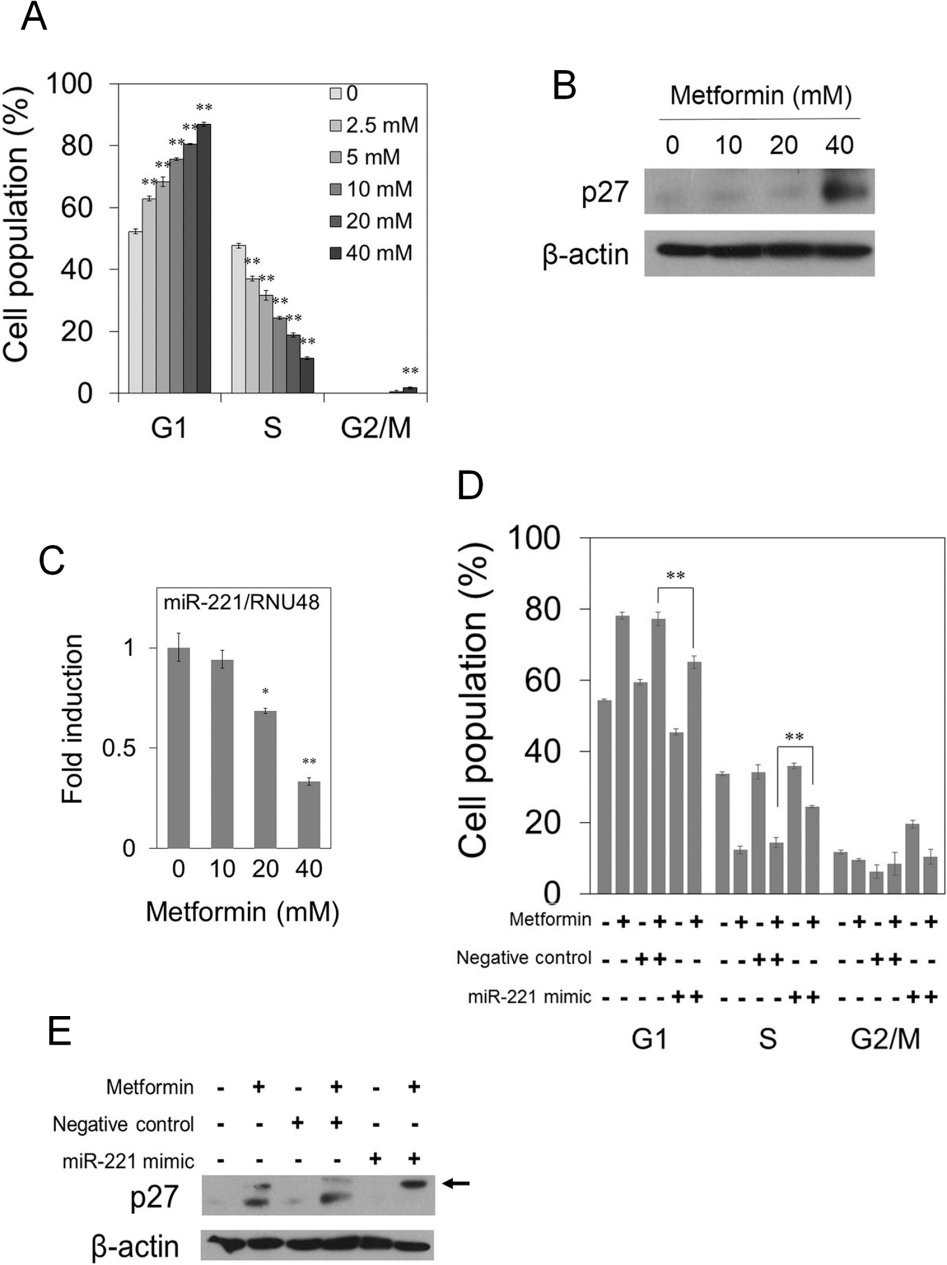
Metformin induces G1-phase arrest in PANC-1 cells through down-regulation of miR-221. (A) PANC-1 cells were treated with the indicated concentrations of metformin for 48 hours. The percentage of cells in each phase of the cell cycle was determined by flow cytometry. (B), (C) PANC-1 cells were treated with the indicated concentrations of metformin for 48 hours. (B) Western blotting for p27. β-actin is a loading control. (C) Real-time RT-PCR quantification of miR-221 expression. The internal control was RNU48. Values are fold change in the expression of miR-221/RNU48 compared with untreated control. (D), (E) PANC-1 cells were transfected with 5 nM miR-221 mimic or 5 nM negative control. After 24 hours, the cells were incubated with or without 40 mM metformin for 48 hours. (D) The percentage of cells in each phase of the cell cycle was determined by flow cytometry. (E) Western blotting for p27. β-actin is a loading control. Arrow, a nonspecific band. Data are the means ± SD of 3 determinations. **P<0.01.

We next performed cell cycle analysis using flow cytometry after treatment with the indicated concentrations of metformin for 48 hours in human pancreatic cancer cells. As shown in Fig 2A, and S1, S2 and S4 Figs, metformin caused G1-phase arrest in a dose-dependent manner. Moreover, we examined the effect of metformin on the expression of G1 phase-related proteins. As a result, metformin at 40 mM increased the expression of p27 protein (Fig 2B). Lee *et al*. previously identified a number of microRNAs with increased expressions, including miR-21, -221, -301, -376a, -155, as well as others in human pancreatic cancer cells [32]. In addition, p27 protein was down-regulated by miR-221 in human pancreatic cancer cells [37]. Therefore, we examined the effect of metformin on the expression of miR-221. As shown in Fig 2C, metformin reduced the expression of miR-221 in a dose-dependent manner.

To examine whether the change in the expression of miR-221 is associated with p27 induction and G1-phase arrest by metformin treatment, we transfected PANC-1 cells with a miR-221 mimic before metformin treatment and performed flow cytometry and Western blot analysis. The transfection of miR-221 mimic reduced the cell population in the G1 phase and the expression of p27 protein induced by metformin, although the G1 phase population in metformin-untreated cells was also decreased (Fig 2D and 2E; S3 Fig). Collectively, these results suggest that the G1-phase arrest induced by metformin at 40 mM may be caused by the induction of p27 partially through the down-regulation of miR-221.

### Metformin enhances TRAIL-induced apoptosis in TRAIL-resistant pancreatic cancer cells

**Fig 3:**
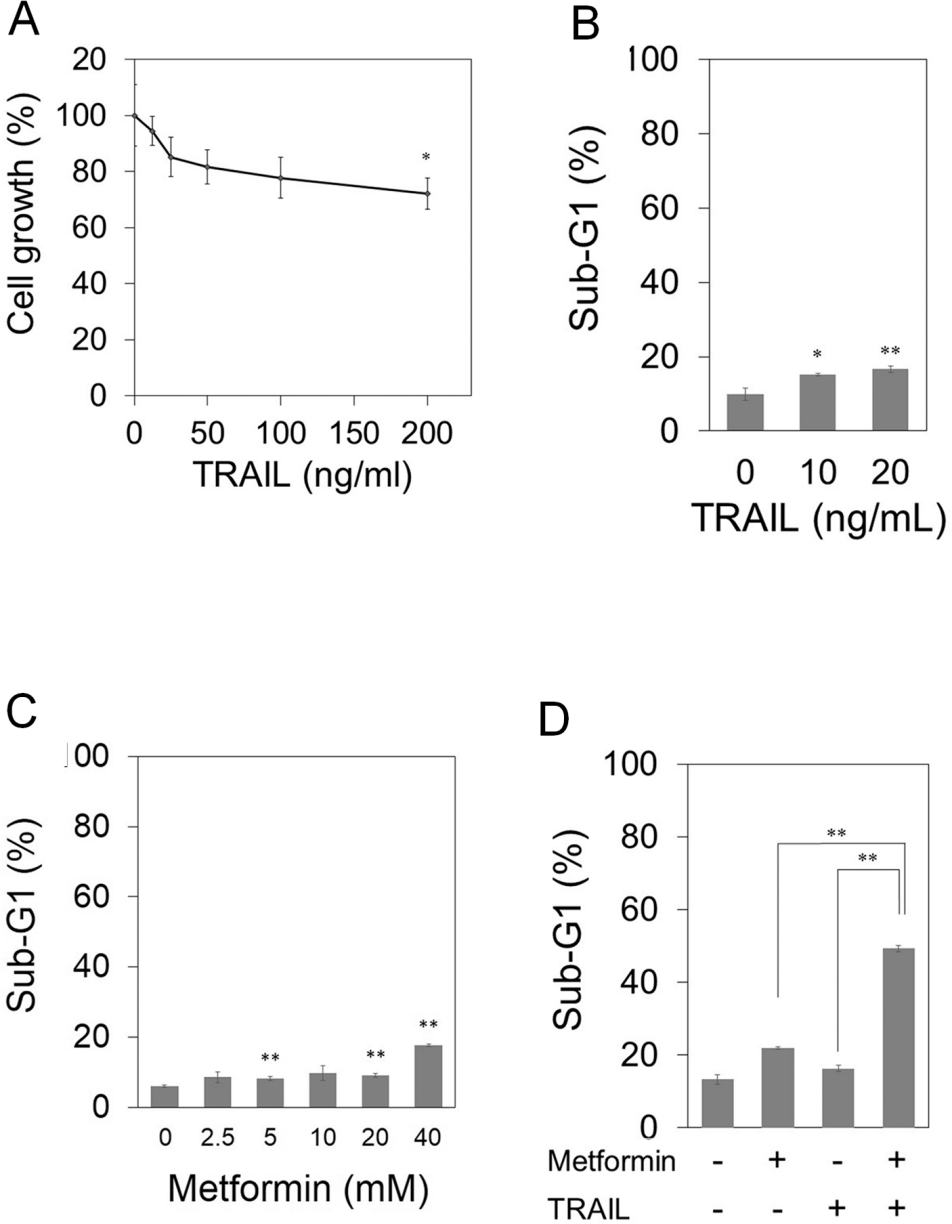
Metformin sensitizes TRAIL-resistant PANC-1 cells to TRAIL-induced apoptosis. (A) PANC-1 cells were treated with the indicated concentrations of TRAIL. After incubation for 72 hours, viable cells were evaluated using a Cell Counting Kit-8. (B), (C) PANC-1 cells were treated with the indicated concentrations of TRAIL (B) or metformin (C) for 48 hours. Sub-G1 populations were analyzed by flow cytometry. (D) Combined effects of 40 mM metformin and/or 10 ng/mL TRAIL for 48 hours. Sub-G1 populations were analyzed by flow cytometry. Data are the means ± SD of 3 determinations. *P<0.05, **P<0.01.

Recently, it was reported that up-regulation of death receptor 5 (DR5) by metformin enhanced TRAIL-induced apoptosis of p53 wild-type malignant tumor cells [34]. Therefore, we examined the effect of metformin on TRAIL sensitization in p53-mutant and TRAIL-resistant pancreatic cancer cells. Three cell lines, PANC-1, AsPC-1, and MIA PaCa-2, were tested for their susceptibility to TRAIL and/or metformin. At first, we treated the cells with exogenous recombinant human TRAIL at the indicated concentrations for 72 hours, and viable cells were counted by a WST-8 assay. Growth of the cell lines was not markedly inhibited with TRAIL (Fig 3A; S4A Fig). We next investigated the effect of TRAIL or metformin on apoptosis induction by measuring the sub-G1 population. Metformin or TRAIL alone slightly induced apoptosis in these three pancreatic cancer cell lines (Fig 3B and 3C; S4B and S4C Figs). However, the combined treatment with metformin and TRAIL markedly increased apoptosis in all tested cell lines (Fig 3D; S4B and S4C Figs).

### Metformin up-regulates the expression of DR5 in pancreatic cancer cells

**Fig 4:**
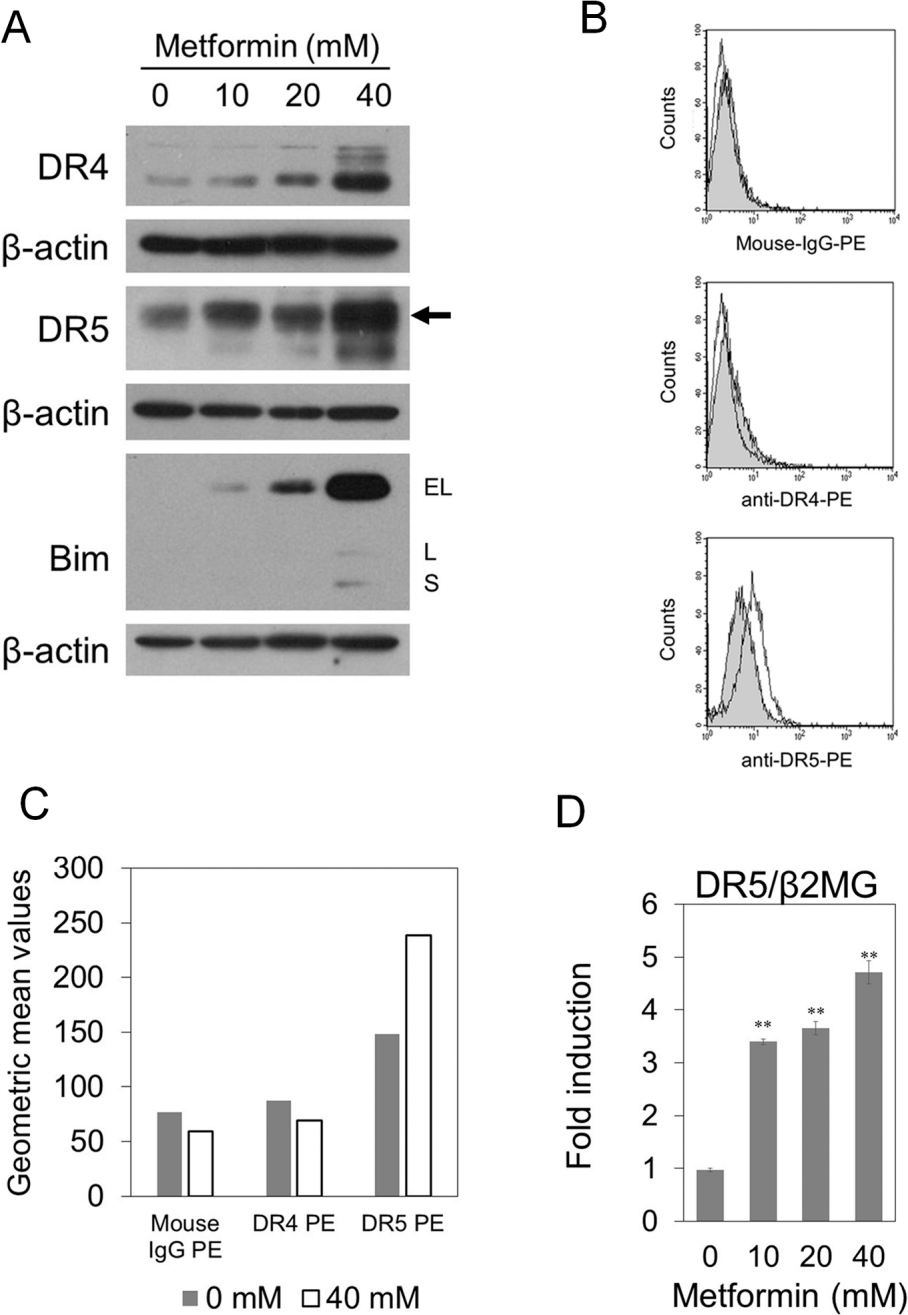
Metformin up-regulates the expression of DR5 in p53 mutant PANC-1 cells. (A) PANC-1 cells were treated with the indicated concentrations of metformin for 48 hours. Western blotting for DR4, DR5 and Bim was performed. β-actin is a loading control. Arrow, nonspecific band. (B), (C) Cell surface expressions of DR4 and DR5 in PANC-1 cells treated with or without 40 mM metformin for 48 hours. Cells were stained with isotype control IgG and monoclonal antibodies against DR4 and DR5. Data were analyzed by flow cytometry. (B) Gray histogram, no treatment; white histogram, metformin treatment. (C) The Y-axis represents the geometric mean values of the cell populations in the histograms. Gray bar, no treatment; white bar, metformin treatment. (D) Quantitative real-time RT-PCR of DR5 mRNA in PANC-1 cells treated with the indicated concentrations of metformin for 48 hours. The internal control was β2MG. Values are fold change in the expression of DR5 mRNA /β2MG mRNA compared with untreated control. Data are the means ± SD of 3 determinations. **P<0.01.

To investigate the mechanisms by which metformin enhances TRAIL-induced apoptosis, we examined the apoptosis-related proteins that were regulated by metformin using Western blot analysis. PANC-1 cells were treated with metformin for 48 hours, and we examined the expression for TRAIL receptors, DR4 and DR5 and several proteins which sensitize cancer cells to TRAIL. As shown in Fig 4A, metformin significantly up-regulated the expression of DR4, DR5 and Bim proteins. We then examined the cell surface expressions of DR4 and DR5 in PANC-1 cells by flow cytometry. Metformin increased the expression of cell surface DR5 only (Fig 4B and 4C). Moreover, we examined the DR5 mRNA level by quantitative real-time RT-PCR after treatment with metformin at the indicated concentrations for 48 hours. As shown in Fig 4D, metformin significantly increased DR5 mRNA expression.

### The enhancement of TRAIL-induced apoptosis by metformin depends on caspases and DR5, but not miR-221

**Fig 5:**
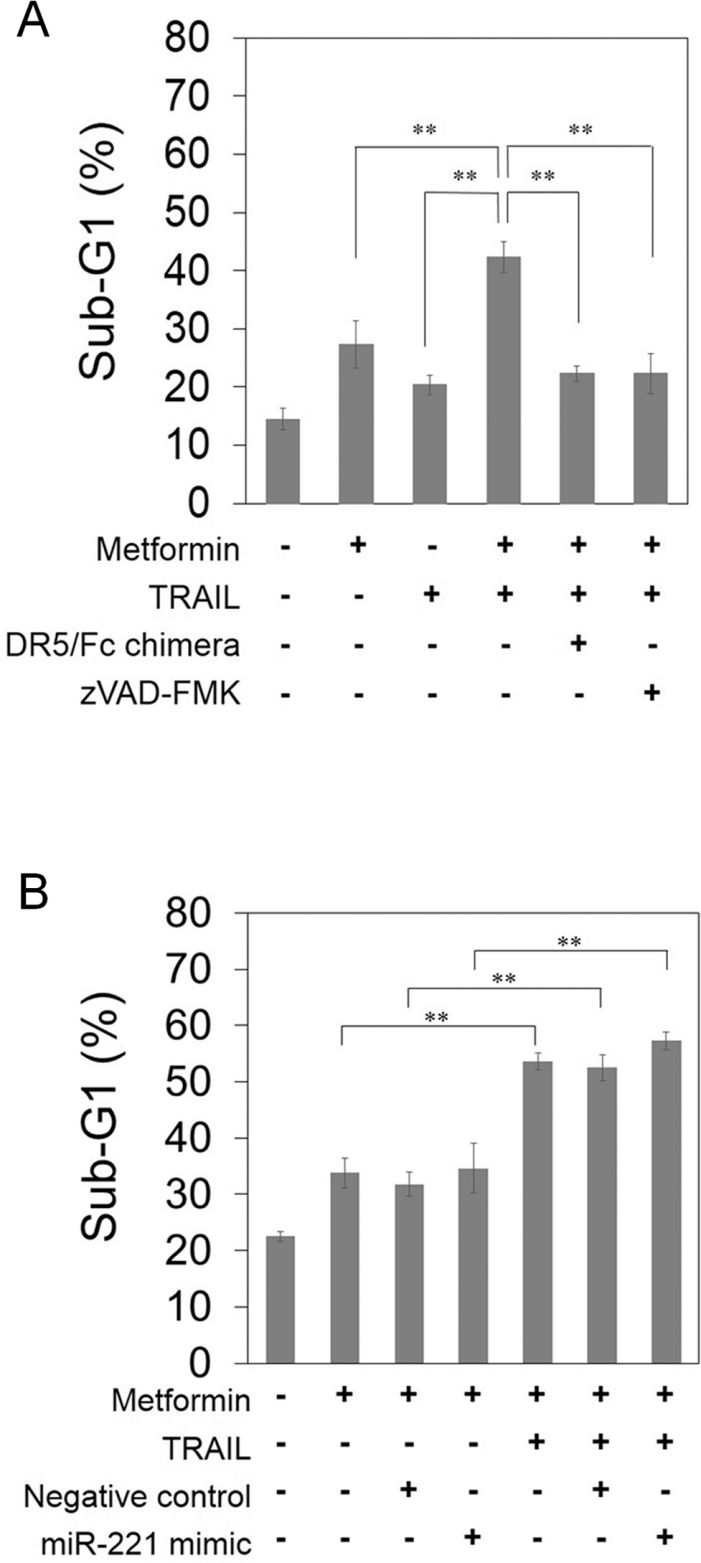
The enhancement of TRAIL-induced apoptosis by metformin depends on caspases and DR5, but not miR-221. (A) PANC-1 cells were treated with 40 mM metformin and/or 10 ng/mL TRAIL for 48 hours with or without the 1 ng/mL DR5/Fc chimera, or the 20 μM zVAD-fmk pan-caspase inhibitor. Sub-G1 populations were analyzed by flow cytometry. (B) PANC-1 cells were transfected with 5 nM miR-221 mimic or 5 nM negative control. After 24 hours, the cells were incubated with or without 40 mM metformin and/or 10 ng/mL TRAIL for 48 hours. Sub-G1 populations were analyzed by flow cytometry. Data are the means ± SD of 3 determinations. **P<0.01.

To analyze the involvement of caspases and DR5 on the TRAIL-induced apoptosis enhanced by metformin in PANC-1 cells, we examined the effect of the pan-caspase inhibitor zVAD-fmk or DR5/Fc chimera protein with dominant negative function against DR5. The apoptosis induced by the combination of metformin and TRAIL was markedly reduced by addition of the DR5/Fc chimera or zVAD-fmk (Fig 5A). These results indicate that TRAIL-induced apoptosis enhanced by metformin was triggered at least in part in a caspase-dependent manner and the interaction between TRAIL and DR5. Next, we tested whether down-regulation of miR-221 by metformin contributed to the TRAIL-induced apoptosis. We transfected PANC-1 cells with a miR-221 mimic before co-treatment with metformin and TRAIL. As shown in Fig 5B, the transfection of miR-221 could not reduce the apoptosis population by co-treatment. These results indicate that miR-221 is not responsible for the enhancement of TRAIL-induced apoptosis by metformin.

## Discussion

Metformin, the “classic” drug for type 2 diabetes, has recently attracted attention as an antitumor agent [5–7]. In addition, our data show novel functions of metformin against pancreatic cancer cells.

It has been reported that metformin induced G1-phase arrest with induction of p27 expression as one of the mechanisms in human cancer cells [16]. Similarly, in our results, metformin induced G1-phase arrest (Fig 2A), and up-regulated p27 protein expression (Fig 2B) in human pancreatic cancer PANC-1 cells. It was reported that down-regulation of miR-221 inhibited the growth of pancreatic cancer cells through up-regulation of PTEN, p27, p57, and PUMA [38]. Among these target molecules of miR-221, p27 is a CDK inhibitor which induces G1-phase arrest in cancer cells [39]. Therefore, we hypothesized that metformin could down-regulate miR-221 expression, resulting in the induction of p27 with G1-phase arrest in pancreatic cancer cells. In this study, we found that metformin down-regulated the expression of miR-221 in pancreatic cancer cells (Fig 2C). Furthermore, both the G1-phase arrest and the induction of p27 by metformin were suppressed by a miR-221 mimic in pancreatic cancer cells (Fig 2D and 2E). From the results, we show for the first time that metformin-induced G1-phase arrest is at least partially caused by p27 induction through down-regulation of miR-221. Meanwhile metformin decreased expressions of cyclin D1 and CDK4 proteins at 10 mM or more (S5 Fig), consistent with previous reports [16, 40]. Therefore, the down-regulation of cyclin D1 and CDK4 protein expressions might contribute to G1-phase arrest by metformin at lower doses. It was reported that miR-221, one of the most well-known OncomiRs, was up-regulated in multiple malignancies including pancreatic cancer [32]. Therefore, miR-221 is considered to be an attractive target for selective treatment against cancer [27–29]. Interestingly, the previous study showed that miR-221 was elevated in the internal mammary arteries of subjects with type 2 diabetes and there was a significant inverse correlation between the oral dose of metformin and the level of miR-221 [33], raising the possibility that our results may be physiological.

On the other hand, previous studies reported that miR-221 also contributed to TRAIL resistance in human cancer cells [28–31]. We therefore hypothesized that metformin may be able to improve the sensitivity of TRAIL via down-regulation of miR-221 in pancreatic cancer cells. To confirm this hypothesis, we first examined the effect of metformin on TRAIL sensitivity in human pancreatic cancer cells. In human pancreatic cancer PANC-1 cells (Fig 3D), AsPC-1 cells (S4B Fig) and MIA PaCa-2 cells (S4C Fig), metformin enhanced the sensitivity of TRAIL. We next examined whether miR-221 was involved in the enhancement of TRAIL sensitivity by metformin. In the present study, the miR-221 mimic did not suppress the apoptosis induced by the combination of metformin and TRAIL human pancreatic cancer PANC-1 cells (Fig 5B). As the possible reason of the difference from previous studies [28–31], we speculate that the anti-apoptotic pathway from miR-221 might not exist in human pancreatic cancer cells tested. Therefore, it is suggested that down-regulation of miR-221 by metformin is involved in G1-phase arrest, but not the apoptosis mentioned above.

We then analyzed the molecular mechanisms enhancing TRAIL-sensitivity, and found that metformin induced the expression of DR5, one of the TRAIL receptors (Fig 4; S6 Fig), and the expression of Bim (Fig 4A). There are no reports that metformin up-regulated the expression of Bim protein in human cancer cells. Bim has a pro-apoptotic function in the downstream of the TRAIL-DR5 pathway. The up-regulation of Bim was also reported to be responsible for enhancement of TRAIL sensitivity [41]. Our data therefore suggest that DR5 and Bim up-regulation by metformin may contribute to sensitization of TRAIL-induced apoptosis.

Truong *et al*., showed that metformin up-regulated DR5 via a p53-dependent pathway [34]. In contrast, our present data have clearly shown that metformin up-regulated DR5 in p53-mutant pancreatic cancer PANC-1 (Fig 4), AsPC-1 and MIA PaCa-2 cells (S6 Fig), indicating that metformin up-regulates DR5 expression in a p53-independent manner. In addition, the apoptosis induced by the combination of metformin and TRAIL was markedly reduced by the DR5/Fc chimera (Fig 5A), indicating that the enhanced TRAIL sensitivity caused by metformin was at least partially DR5 dependent. Interestingly, Ozawa *et al*. demonstrated that the expression levels of DR5 protein in pancreatic cancer samples were 5.1-fold higher (P<0.01) than the normal pancreatic tissue [24].

It has been reported that metformin has various functions [6, 7, 10–16]. Recently, clinical trials of combinations with metformin and various anticancer agents are ongoing from the viewpoint of drug repositioning [8, 9]. In the previous study, Gritti *et al*. showed that 40 mM metformin did not affect the viability of human umbilical cord-derived mesenchymal stem cells, and described that metformin specifically elicits antitumoral effects without interfering with normal cell viability [42]. We demonstrate here that the combination of metformin and TRAIL is very effective against human pancreatic cancer cells, raising the possibility of a combination strategy in the treatment of pancreatic cancer.

## Supporting Information

S1 Fig Metformin induces G1-phase arrest in PANC-1 cells. (A), (B) The representative histograms of Fig 2A. (A) no treatment. (B) 40 mM metformin.
(TIF)

S2 Fig Metformin induces G1-phase arrest in MIA PaCa-2 cells. MIA PaCa-2 cells were treated with the indicated concentrations of metformin for 24 hours. The percentage of cells in each phase of the cell cycle was determined by flow cytometry. Data are the means ± SD of 3 determinations. *P<0.05, **P<0.01.
(TIF)

S3 Fig Metformin induces G1-phase arrest in PANC-1 cells through down-regulation of miR-221. The representative histograms of Fig 2D. (A) Mock. (B) 40 mM metformin. (C) mimic negative control. (D) mimic negative control and 40 mM metformin. (E) miR-221 mimic. (F) miR-221 mimic and 40 mM metformin.
(TIF)

S4 Fig Metformin sensitizes AsPC-1 and MIA PaCa-2 pancreatic cancer cells to TRAIL. (A) AsPC-1 cells were treated with the indicated concentrations of TRAIL. After incubation for 72 hours, viable cells were evaluated using a Cell Counting Kit-8. (B) AsPC-1 cells were treated with the 10 ng/mL TRAIL and/or 40 mM metformin for 48 hours. (C) MIA PaCa-2 cells were treated with the 4 ng/mL TRAIL and/or 40 mM metformin for 24 hours. Sub-G1 populations were analyzed by flow cytometry. Data are the means ± SD of 3 determinations. *P<0.05, **P<0.01.
(TIF)

S5 Fig Metformin down-regulates the expressions of cyclin D1 and CDK4. PANC-1 cells were treated with the indicated concentrations of metformin for 48 hours. Western blotting for cyclin D1 and CDK4 was performed. β-actin is a loading control.
(TIF)

S6 Fig Metformin up-regulates DR5 proteins in p53 mutant pancreatic cancer cells. (A) AsPC-1 cells were treated with the indicated concentrations of metformin for 48 hours. (B) MIA PaCa-2 cells were treated with the indicated concentrations of metformin for 24 hours. Western blotting for DR5 was performed. β-actin is a loading control.
(TIF)
