# Supplementary figures and images for: Metformin Causes G1-Phase Arrest via Down-Regulation of MiR-221 and Enhances TRAIL Sensitivity through DR5 Up-Regulation in Pancreatic Cancer Cells
Source: PLoS One. 2015 May 8;10(5):e0125779. doi: 10.1371/journal.pone.0125779 (PMC4425682; doi:10.1371/journal.pone.0125779)

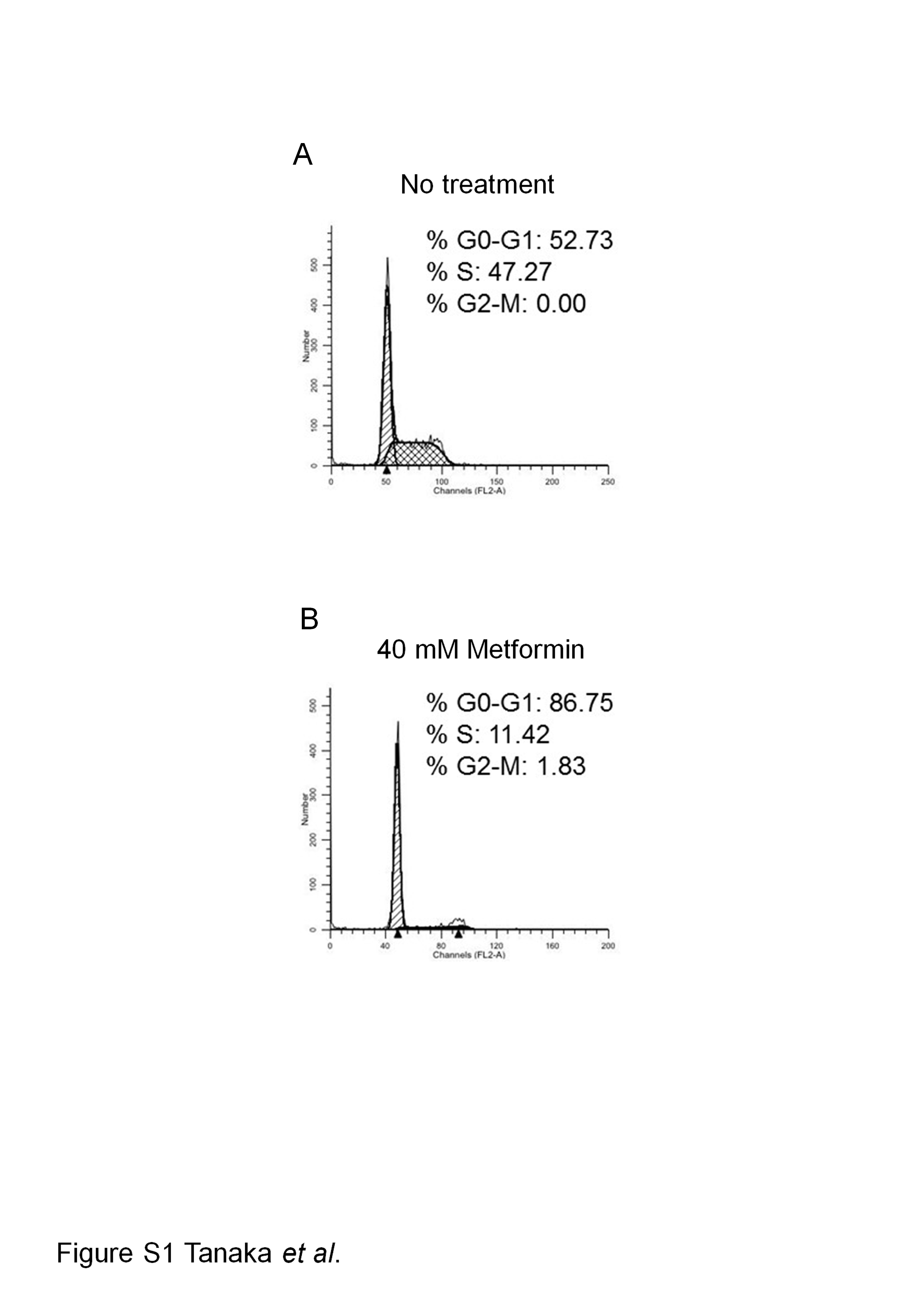

Supplement: S1 Fig — (A), (B) The representative histograms of Fig 2A. (A) no treatment. (B) 40 mM metformin. (TIF) [file pone.0125779.s001.tif]

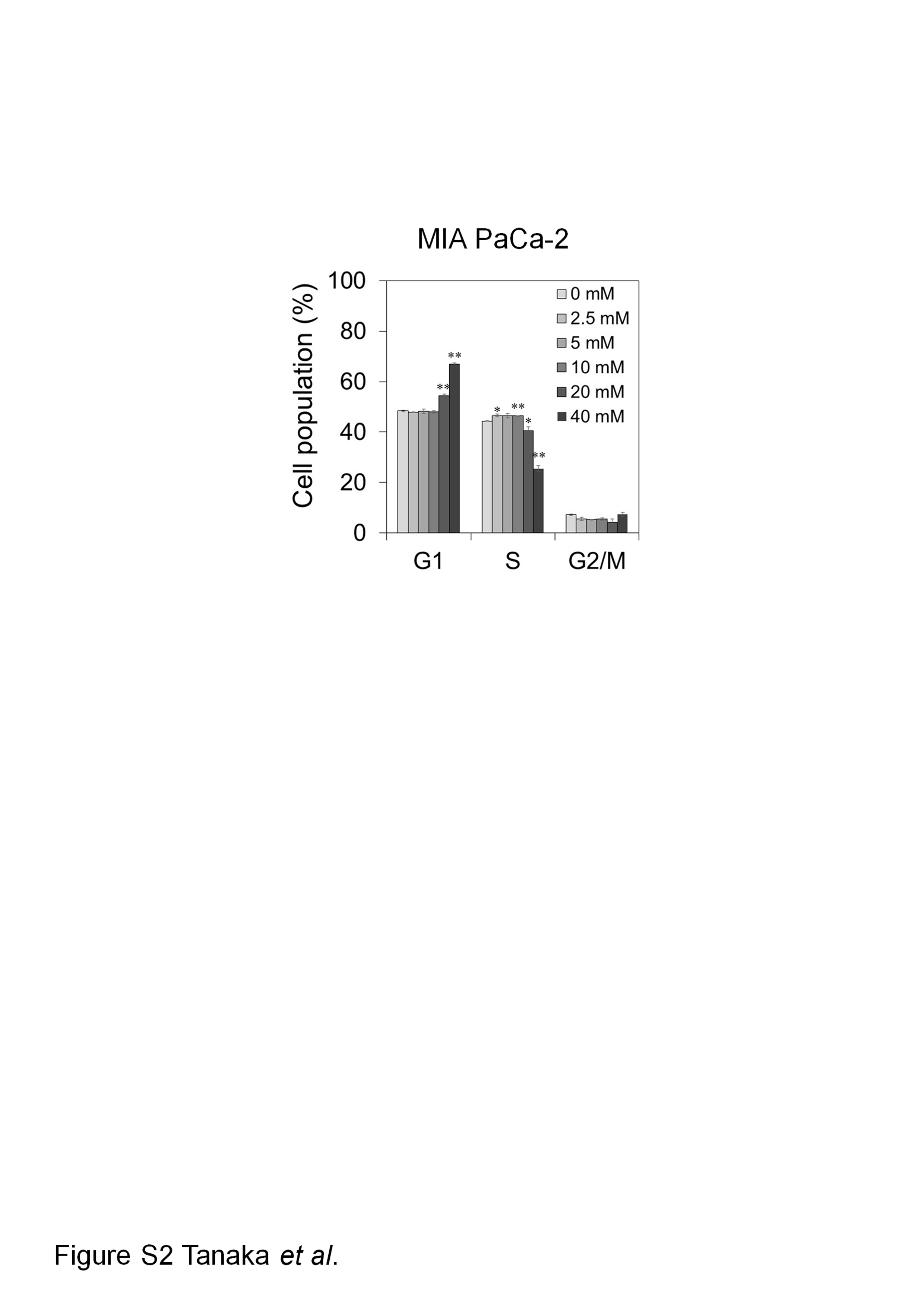

Supplement: S2 Fig — MIA PaCa-2 cells were treated with the indicated concentrations of metformin for 24 hours. The percentage of cells in each phase of the cell cycle was determined by flow cytometry. Data are the means ± SD of 3 determinations. *P<0.05, **P<0.01. (TIF) [file pone.0125779.s002.tif]

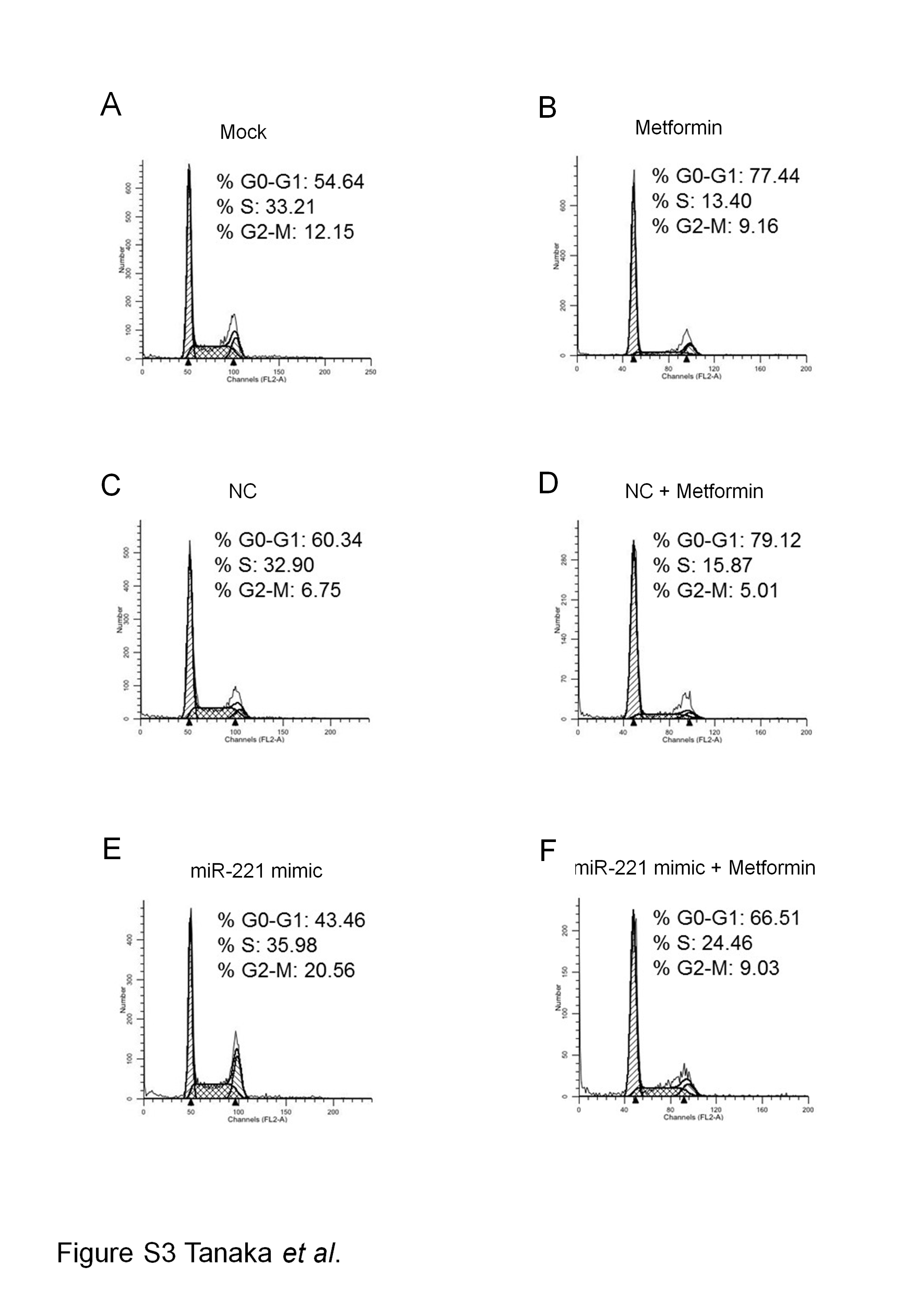

Supplement: S3 Fig — The representative histograms of Fig 2D. (A) Mock. (B) 40 mM metformin. (C) mimic negative control. (D) mimic negative control and 40 mM metformin. (E) miR-221 mimic. (F) miR-221 mimic and 40 mM metformin. (TIF) [file pone.0125779.s003.tif]

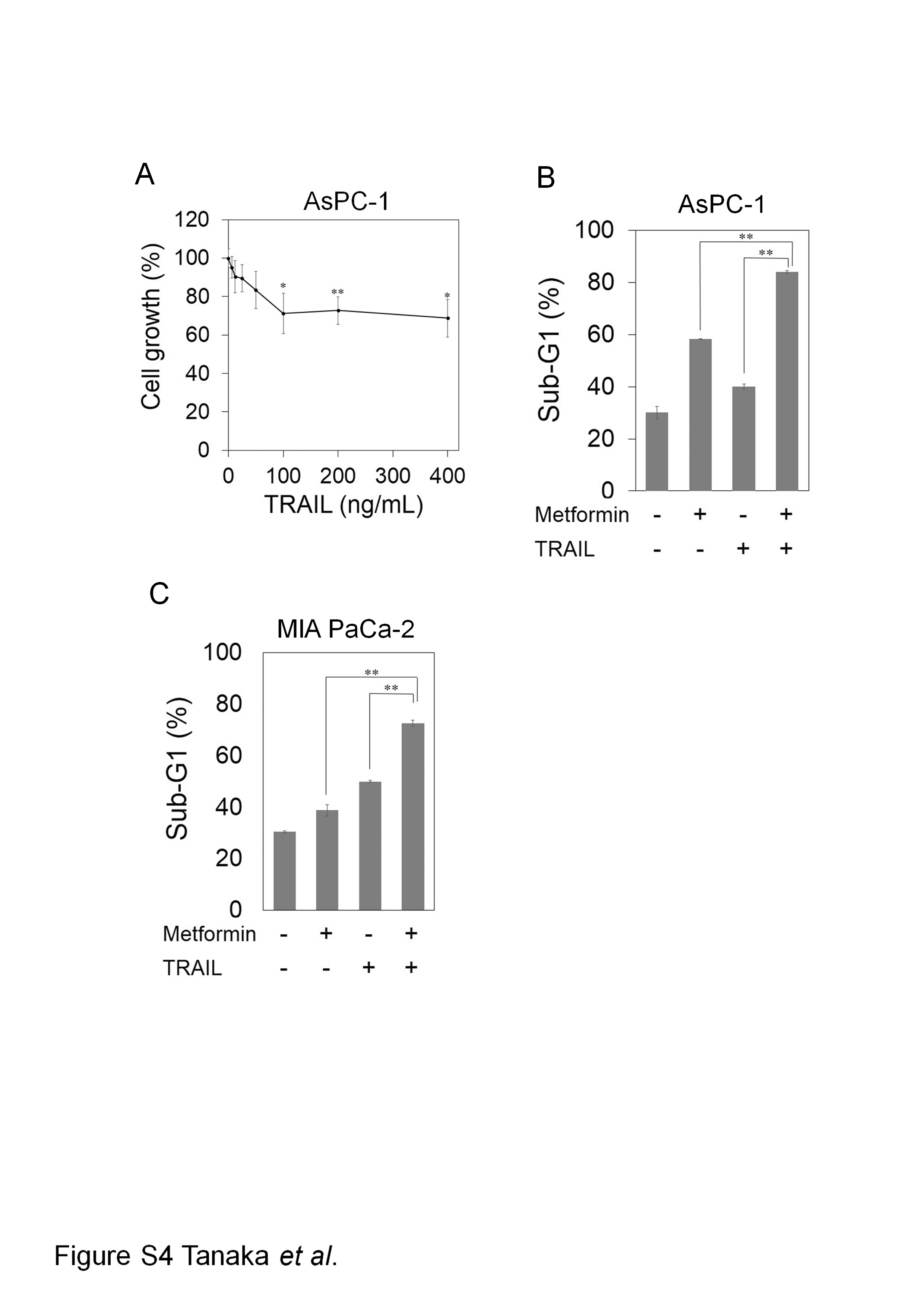

Supplement: S4 Fig — (A) AsPC-1 cells were treated with the indicated concentrations of TRAIL. After incubation for 72 hours, viable cells were evaluated using a Cell Counting Kit-8. (B) AsPC-1 cells were treated with the 10 ng/mL TRAIL and/or 40 mM metformin for 48 hours. (C) MIA PaCa-2 cells were treated with the 4 ng/mL TRAIL and/or 40 mM metformin for 24 hours. Sub-G1 populations were analyzed by flow cytometry. Data are the means ± SD of 3 determinations. *P<0.05, **P<0.01. (TIF) [file pone.0125779.s004.tif]

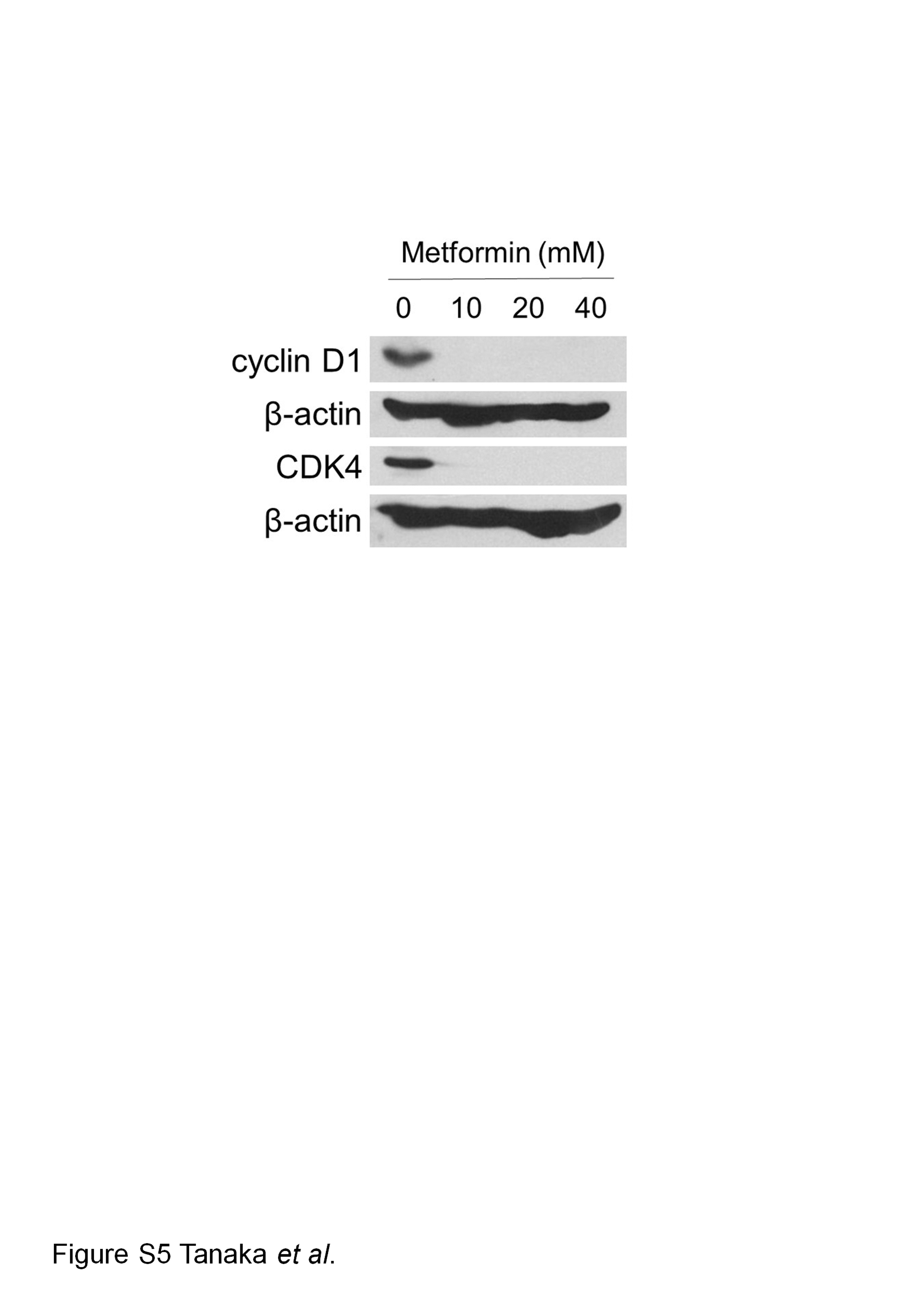

Supplement: S5 Fig — PANC-1 cells were treated with the indicated concentrations of metformin for 48 hours. Western blotting for cyclin D1 and CDK4 was performed. β-actin is a loading control. (TIF) [file pone.0125779.s005.tif]

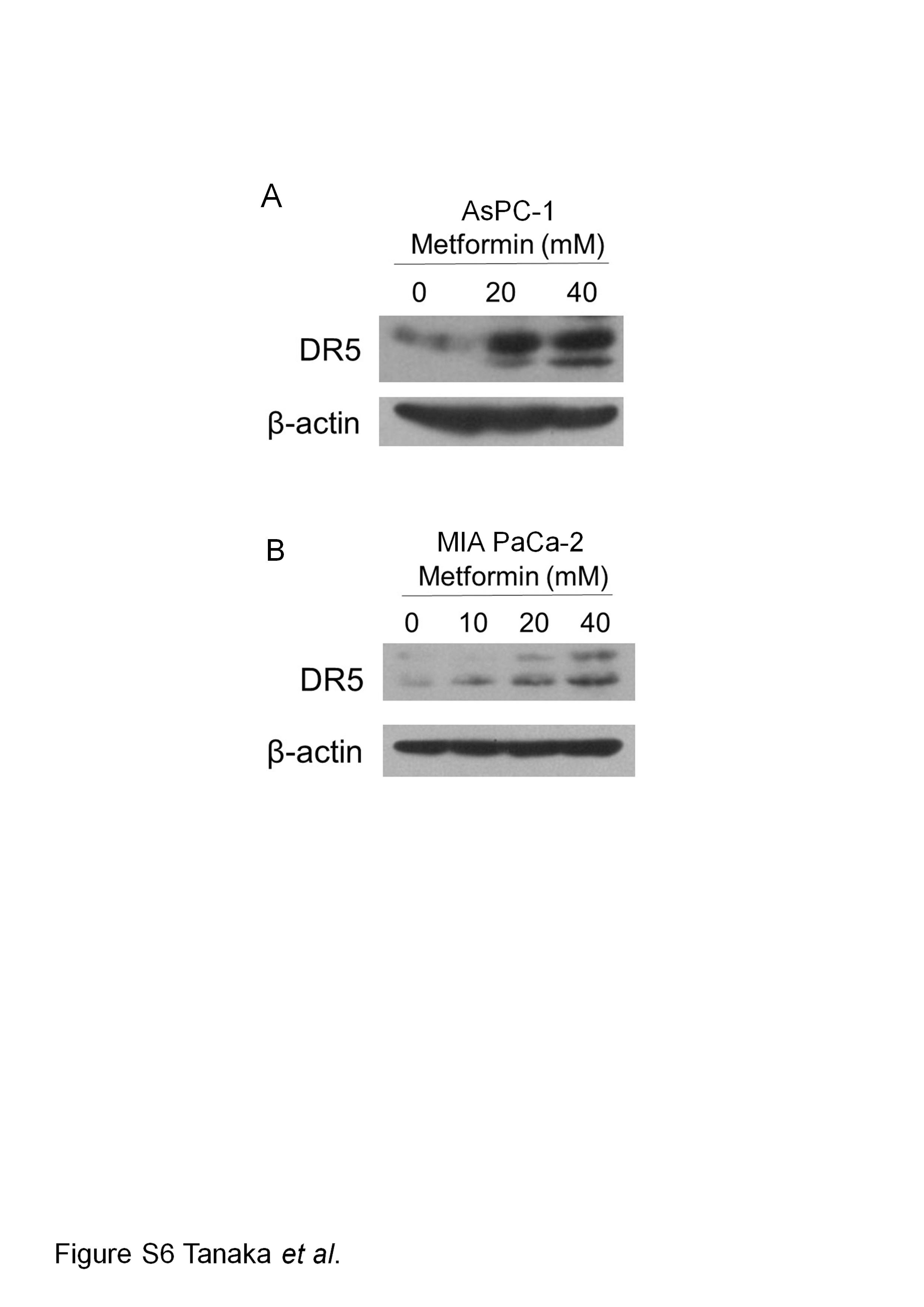

Supplement: S6 Fig — (A) AsPC-1 cells were treated with the indicated concentrations of metformin for 48 hours. (B) MIA PaCa-2 cells were treated with the indicated concentrations of metformin for 24 hours. Western blotting for DR5 was performed. β-actin is a loading control. (TIF) [file pone.0125779.s006.tif]
